# Changes in EEG Activity and Cognition Related to Physical Activity in Older Adults: A Systematic Review

**DOI:** 10.3390/life14040440

**Published:** 2024-03-26

**Authors:** Luis Miguel Rodríguez-Serrano, Marina Wöbbeking-Sánchez, Lizbeth De La Torre, Ruben Pérez-Elvira, María Elena Chávez-Hernández

**Affiliations:** 1 Facultad de Psicología, Universidad Anáhuac México, Universidad Anáhuac Avenue 46, Lomas Anáhuac, Huixquilucan 52786, Mexico; l.rodriguez@anahuac.mx (L.M.R.-S.); mariele_chavez@yahoo.com (M.E.C.-H.); 2 Facultad de Psicología, Universidad de Salamanca, Avenida de la Merced 109, 37005 Salamanca, Spain; 3 Facultad de Psicología, Universidad Pontificia de Salamanca, Calle de la Compañía 5, 37002 Salamanca, Spain; ldelatorrelo@upsa.es; 4 Laboratorio de Neuropsicofisiología, NEPSA Rehabilitación Neurológica, Facultad de Psicología, Universidad Pontificia de Salamanca, Calle de la Compañía 5, 37002 Salamanca, Spain

**Keywords:** electroencephalography, physical activity, cognition, older adults

## Abstract

Aging is generally associated with a decline in important cognitive functions that can be observed in EEG. Physical activity in older adults should be considered one of the main strategies to promote health and prevent disease in the elderly. The present study aimed to systematically review studies of EEG activity and cognitive function changes associated with physical activity in older adults. Records from PubMed, Scopus, and EBSCO databases were searched and, following the PRISMA guidelines, nine studies were included in the present systematic review. A risk of bias assessment was performed using the National Institute of Health Quality Assessment Tool for Case-control Studies instrument. The studies analyzed used two main strategies to determine the effects of physical activity on cognition and EEG: (1) multiscale entropy and power frequencies; and (2) event-related potentials. In terms of EEG activity, it can be concluded that exercise-induced neuroplasticity underlies improvements in cognitive function in healthy older adults.

## 1. Introduction

Nowadays, the proportion of the population aged 65 years and older is increasing throughout the world [1]; this population is therefore at increased risk of cognitive impairment and dementia [2]. In this regard, aging is a progressive process in which changes are observed in different domains, such as cognitive, psychological, physical, and social. Nevertheless, these can sometimes show a pathological tendency that causes accentuated changes of deterioration, ending in important disorders such as mild cognitive impairment or neurodegenerative diseases [3,4]. Longevity is increasing in our society due to various factors [5]. The World Health Organization (WHO) [6] states that aging is caused by an accumulation of molecular and cellular damage over time, resulting in a progressive decline of physical and mental abilities and increasing the risk of disease and death. Due to this demographic revolution, society must be prepared to care for the elderly in a multidisciplinary way, as more advanced ages are being reached every day. This phenomenon is leading to a higher prevalence of neurodegenerative diseases which are becoming one of the largest clinical, public health, and social challenges of our time [7,8].

The WHO [6] defines physical activity (PA) as body movements generated by skeletal muscles that consume energy. Moderate to vigorous intensity PA has been shown to have health benefits, including improvements in cardiovascular and bone health, as well as mental health, cognitive vitality, reductions in obesity, depression, and subjective well-being and self-esteem [9,10]. One of the relevant aspects of aging that maintains the quality of life of older adults is the practice of PA [11]. According to the WHO, it is recommended that older adults incorporate aerobic, strength, and balance exercises to reduce the risk of falls [12]. Additionally, in older adults, PA has been shown to reduce social isolation and improve quality of life [13,14] and cognitive function; it is also associated with positive changes in brain electrical activity [9]. Furthermore, studies have shown that older adults with higher cognitive, physical, and motivational reserves have less cognitive decline [15], and that there is a relationship between PA and cognition [16,17,18].

Electroencephalography (EEG) is one of the most widely used neurophysiological techniques to study brain function in aging [19,20]. EEG records brain electrical activity with high temporal resolution; it is used to map brain activity and to reveal how this relates to cognitive processes [21]. EEG also records rhythmic patterns of neuronal activity or neuronal oscillations that can be analyzed using spectral analysis and event-related potentials (ERP) techniques. For example, quantitative electroencephalography (qEEG) and multiscale entropy (MSE) analyses help evaluate the internal entropy associated with specific frequency levels (alpha, beta, and delta functions), as well as how this entropy differs from the norm [22]. MSE provides an index of dynamic changes in intrinsic brain activity and “meaningful structural richness” for a biological system with underlying chaotic dynamics [23]. qEEG measures brain activity, usually at rest, and is sensitive to changes in synaptic function and connectivity [24]. In addition, ERP captures task-based and time-locked averaged brain responses [24]. ERP registers the electrical field generated by populations of neurons in response to an event and records brain activity during a task; brain waves are then amplified, digitized, and filtered to produce an average waveform with positive (P) or negative (N) slopes, resulting in ERP amplitudes such as P50, P200, P300, N100, and N200 latencies [24,25]. Moreover, ERP is a very useful measure for linking neural function to cognitive processes, particularly for assessing synaptic dysfunction [24].

EEG neural oscillations have been used to identify brain changes in older adults. In this regard, two models of changes in neural and brain activation patterns in aging have been studied: the posterior–anterior shift in aging (PASA) model and the hemispheric asymmetry reduction in older adults (HAROLD) model. The PASA model suggests that frontal over-engagement occurs because the functional integrity of posterior regions declines with age, whereas the HAROLD model suggests that prefrontal activation during cognitive task performance is less lateralized in older adults compared to young adults [26] (for a detailed review on these and other models of aging, see Grandi and Tirapu Ustárroz [27]). Furthermore, a global slowing of brain activation is associated with age-related changes in brain activity; however, this global slowing is difficult to demonstrate by changes in neurotransmission and conduction velocity [28]. Signs of aging in resting EEG/MEG include (1) a significant reduction in amplitude of alpha activity (8–13 Hz), (2) a slowdown of background activity (dominant alpha rhythm), and (3) a global power increase and topographic delta location (1–4 Hz) and theta (4–8 Hz) frequency [29,30]. In the same sense, attenuated spontaneous gamma oscillations [31], a posterior-to-anterior shift of oscillatory activity, increasing frontal activity, and decreasing occipital activity have been reported [26].

In healthy individuals, aging is associated with impairments in cognitive information processing and an average cognitive decline in attention, memory, and other cognitive functions [26,29]. These changes can be seen in the EEG from back to front areas and can also be observed as reduced hemispheric asymmetry during cognitive task performance [26]. Furthermore, abnormal aging studies using qEEG have associated the preclinical and early clinical stages of Alzheimer’s disease with disrupted resting functional connectivity between different brain regions [32]. Given the importance and benefits of PA in aging, such as improved cognitive function, we conducted a systematic review to identify findings in studies from healthy humans in terms of EEG measurements and cognition as they relate to physical activity in older adults.

## 2. Materials and Methods

### 2.1. Information Sources and Search Strategy

Following the format of the PICO (population, intervention, comparison, outcome) research question, the elements specified in the present systematic review are as follows: Population: older adults; Intervention: physical activity; Comparison: control or sedentary group, or pre/post measure; and Outcome measure: effect on EEG and cognition.

A systematic search was also conducted following the PRISMA guidelines for systematic reviews and meta-analyses [33,34]. Three databases were searched: PubMed, EBSCO, and Scopus. The following Medical Subject Headings (MeSH) terms were used for the search: “physical activity”, “exercise”, “sedentary behavior”, “EEG”, “electroencephalography”, “aging”, “healthy aging”, “cognition”. These descriptors were searched in the title and abstract fields.

English-language scientific articles were included without restrictions on publication date. Inclusion criteria were peer-reviewed articles or longitudinal and/or cross-sectional studies. Review articles and systematic reviews and/or meta-analysis articles were excluded, as were cases in which the full text was not available.

### 2.2. Data Extraction and Assessment of Risk of Bias

All studies were extracted by two authors (R-S and C-H) and reviewed by two other authors (W-S and D-L). The identification and extraction of the articles for this systematic review were performed using PRISMA. In addition, the risk of bias was assessed by two reviewers (R-S and C-H) using the National Institute of Health (NIH) Quality Assessment of case-control studies tool; this process was then reviewed by two authors (W-S and D-L).

## 3. Results

### 3.1. Search Results

**Figure 1:**
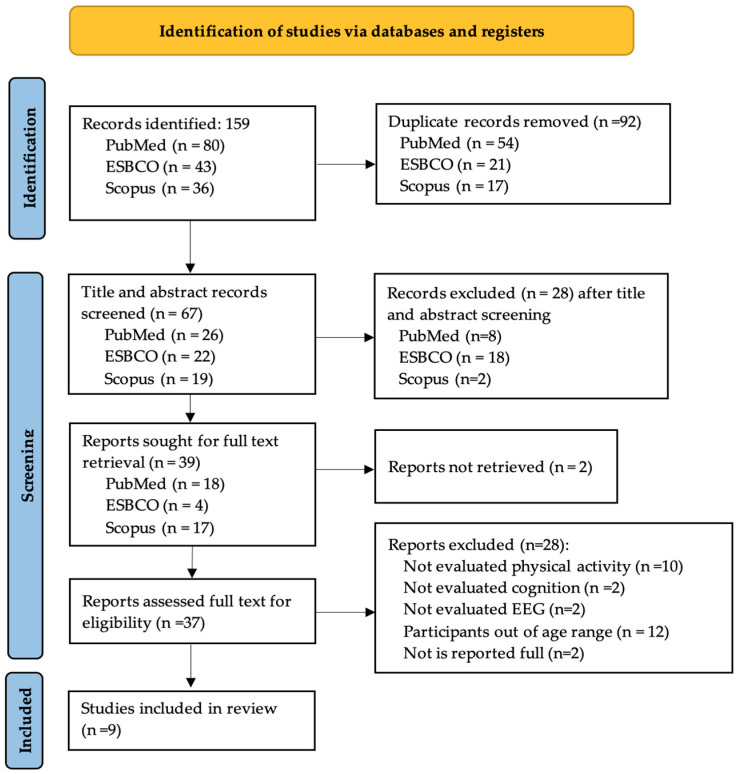
Flow diagram of study selection.

A total of 159 studies were identified from three databases; 92 duplicates were removed, and 67 records were included after screening for titles and abstracts. Of these, 39 were included if the full text was available. After full-text screening, 9 studies were eligible for inclusion in the present systematic review (Figure 1).

### 3.2. Risk of Bias Assessment

The results of the risk of bias assessment are presented in Table 1. The risks of selection bias, performance bias, detection bias, attrition bias, information bias, and others were assessed and grouped into the following twelve questions included in the NIH Quality Assessment of Case-Control Studies Tool (https://www.nhlbi.nih.gov/health-topics/study-quality-assessment-tools, accessed on 15 December 2023):

Q1: Was the research question or objective in this paper clearly stated and appropriate?
Q2: Was the study population clearly specified and defined?
Q3: Did the authors include a sample size justification?
Q4: Were controls selected or recruited from the same or similar population that gave rise to the cases (including the same timeframe)?
Q5: Were the definitions, inclusion and exclusion criteria, algorithms or processes used to identify or select cases and controls valid, reliable, and implemented consistently across all study participants?
Q6: Were the cases clearly defined and differentiated from controls?
Q7: If less than 100 percent of eligible cases and/or controls were selected for the study, were the cases and/or controls randomly selected from those eligible?
Q8: Was there use of concurrent controls?
Q9: Were the investigators able to confirm that the exposure/risk occurred prior to the development of the condition or event that defined a participant as a case?
Q10: Were the measures of exposure/risk clearly defined, valid, reliable, and implemented consistently (including the same time period) across all study participants?
Q11: Were the assessors of exposure/risk blinded to the case or control status of participants?
Q12: Were key potential confounding variables measured and adjusted statistically in the analyses? If matching was used, did the investigators account for matching during study analysis?

**Table 1:** Risk of bias assessment using NIH Quality Assessment of Case-Control Studies.

Study	Q1	Q2	Q3	Q4	Q5	Q6	Q7	Q8	Q9	Q10	Q11	Q12
Oken, 2006 [35]	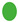	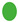	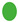	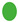	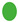	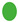	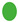	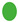	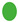	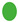	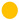	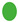
Wang, 2014 [23]	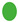	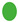	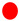	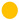	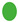	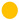	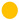	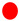	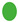	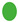	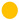	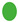
Chuang, 2015 [36]	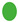	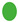	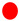	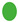	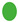	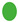	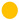	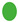	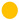	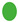	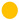	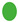
Gajewski, 2015 [37]	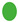	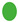	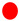	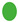	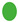	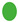	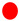	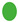	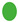	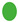	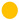	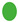
Schättin, 2016 [38]	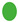	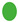	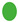	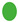	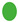	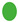	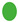	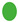	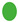	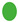	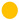	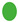
Schättin, 2018 [39]	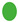	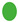	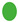	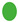	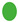	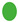	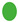	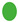	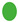	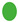	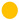	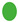
Gajewski, 2018 [40]	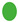	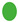	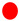	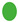	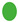	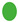	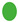	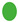	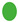	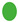	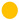	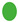
Schättin, 2019 [41]	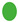	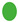	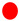	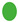	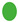	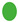	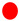	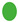	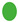	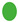	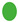	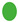
Adcock, 2020 [42]	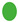	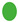	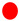	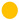	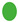	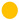	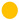	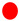	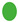	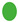	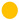	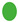

Note: low risk of bias (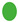); high risk of bias (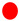); unclear risk of bias (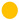).

### 3.3. Physical Activity and Cognition and EEG in Aging

The studies included in this systematic review are listed in Table 2 and were published from 2006 to 2020. All studies included participants over 65 years of age. Six studies included both female and male participants (66.7%), two studies included only females (22.2%), and one study included only males (11.1%). Three studies used an observational design (33.3%), three studies used a randomized controlled trial design (33.3%), two studies used an interventional design (22.2%), and one study used a randomized control trial design (11.1%). Regarding EEG analysis, six studies (66.7%) evaluated EEG (spectral power, quantitative) while three studies (33.3%) evaluated event-related potentials (ERP). Furthermore, six studies applied a PA program, of which five (83%) used a medium-term (7–23 weeks) intervention, while one study (17%) evaluated a long-term (24 weeks or more) intervention.

Of the total number of articles included in this systematic review, five studies (56%) found a trend toward significant improvement in cognitive functions such as attention, executive function, working memory, and visuospatial attention in those who underwent an intervention program with increased physical activity over a long period. At the same time, four studies (44%) also confirmed this improvement using EEG techniques to monitor changes at a neurological level in participants [23,36,37,39,42].

As reported in the results of the included analyses, no significant differences in improved outcomes were found by sex. However, there was a slight trend that may indicate that in the groups where most of the participants were men, there was an improvement in both general cognitive status and EEG assessments.

#### 3.3.1. Effects of the Physical Activity on Cognition and EEG: Multiscale Entropy, Power Frequency

Aging is associated with changes in EEG and an expected decline in key cognitive functions. In this regard, evidence from observational studies [23] shows that physical activity improves visuospatial attention and working memory, as evidenced by higher accuracy for the active group compared to inactive participants in a non-delayed/delayed matching-to-sample test, as well as shorter reaction times (RTs) for the attention condition and longer RTs for the working memory condition; in addition, higher sample EEG entropy was found in the physical activity group in the attention condition and the memory retrieval phase in the Fz electrode, while no group differences in EEG were found in the working memory condition, nor was altered EEG activity found in a multiscale entropy analysis. Furthermore, a randomized controlled study showed that comparing pre-and post-test measures in older adult subjects with an exergame intervention showed a significant decrease in RT on working memory, divided attention, go/no go, and set-shifting measures of the Test for Attentional Performance (TAP), while the balance training group showed a significant decline in RT only on the set-shifting measure. The same study also reported a significant decrease in relative EEG theta power over the prefrontal cortex in participants which had undertaken physical activity (exergame) [38]. In this regard, another observational study [39] showed that a physically active lifestyle did not improve cognition but showed significantly higher power activity in alpha frequency at prefrontal-located EEG electrodes, suggesting that higher EEG power in the alpha frequency band and a physically active lifestyle may be associated with the ability to generate long-term potentiation in older adults. In contrast, the results from a randomized controlled trial [41] found that exergame training did not alter cognition and EEG response-locked potentials over time (pre/post) in prefrontal-located channels Fp1 and Fp2. These discrepancies between studies can be attributed to many factors; for example, it has been proved that the study design, the type of EEG analysis performed, and the equipment used can significantly impact the results. Nakamura et al. [43] proved this hypotheses by developing a system to obtain technically satisfactory EEG recordings. This development could contribute positively to the accuracy of EEG interpretation. Those authors observed that difficulties may arise, on one hand, due to the technical and physiological components used, and on the other, when the evaluation is performed in a group of subjects or a single case. All the same, another element that may interfere with the results is the equipment employed. This includes the selection and use of the appropriate EEG equipment for the study, as well as the placement and impedance of the electrodes [44]. In addition, the type of electrodes selected, i.e., whether they are gel or gel-free, is also an important consideration [45]. On the other hand, an interventional study [42] showed that an exergame intervention did not change resting state EEG recorded at a 500 Hz sampling rate in a pre/post comparison but did improve cognition, whereas in a randomized control trial [35], physical activity did not show changes in qEEG measure of alertness (posterior median power frequency) nor in the ability to focus. In that study, attention span and concentration skills were measured by the Stroop Color and Word Test. Taken together, these results suggest that physical activity has neuroprotective effects on brain activity and improves cognition.

#### 3.3.2. Effects of the Physical Activity on Cognition and EEG: Event-Related Potentials

ERPs have a high temporal resolution that is suitable for revealing underlying neurocognitive processes. In this regard, an interventional study by Chuang et al. [36] showed that a 12-week physical activity intervention had effects on ERP latency but not amplitude, compared to a control group, as evidenced by a significantly shorter N2 and P3 latency. Moreover, in a pretest/posttest analysis, the physical activity intervention resulted in smaller posttest RTs during a selective attention task, while the control group showed longer posttest RTs, suggesting that physical activity improves selective attention in older women. Additionally, an observational study by Gajewski and Falkestein [37] showed that low active seniors had generally delayed P2 latency at the Fz electrode compared to active seniors, physically active seniors had greater N2 amplitude at all frontocentral electrodes compared to low active seniors, and the mean amplitude of N450 at the Cz electrode was more negative in physically active seniors compared to low active seniors. In addition, low active seniors showed a significantly higher interference index in the Stroop test compared to physically active seniors. In contrast, a randomized control study [40] compared the effects of 4 months of physical training, relaxation training, cognitive training, and a control group on memory and ERP P3 at Fz (P3a) and Pz (P3b) electrodes. The results showed no significant effects of physical activity interventions on short- and long-term memory and delayed recognition. Additionally, no changes in ERP P3a and P3b associated with physical activity were found.

**Table 2:** Description of studies evaluating EEG activity and cognitive function associated with physical activity in older adults.

Study	Participants Characteristics	EEG	Cognitive Process Evaluated and Task	Physical Activity Program	Results
Oken, 2006 (RCT) [31]	Healthy men and women aged 65–85 years. Yoga group n = 44 Exercise group: n = 47 Control: n = 44	Quantitative-EEG posterior median power frequency	Attention: Stroop Color Alertness: Word Test	Yoga classes (90 min) for 6-month Aerobic intervention: walking for 1 h (1 class per week)	= Yoga and aerobic intervention did not produce improvements in cognitive function = EEG auditory median power frequency
Wang, 2014 (OBS) [23]	Healthy men and women aged 66–70 years. Physically active: n = 24 Physically inactive n = 24	EEG Multiscale entropy analysis	Visuo-spatial attention and working memory	Estimation of physical activity levels with questionnaire; participants to recall physical activity in the past 7 days	↑ visuo-spatial attention and working memory in physically active group ↑ EEG multiscale entropy in physically active group
Chuang, 2015 (INT) [36]	Sedentary females aged 65–75 years. Dance Dance Revolution (DDR): n = 7 Brisk walking (BW): n = 11 Control group: n = 8	EEG-ERP: N2, P3	Selective attention: flanker task	Intervention: three times per week for 3 months, exergame DDR or BW	↑ response speed in congruent and incongruent conditions in the flanker task with DDR and BW intervention ↓ N2 and P3 latencies in DDR and BW groups
Gajewski, 2015 (OBS) [37]	Healthy men aged 65–70 years. Low active: n = 20 Active: n = 20	EEG-ERP: CNV, P2, N2, N450	Attention: D2, Stroop Color, Digit-Symbol-Test Memory: Digit-Span-Test, Speed of processing: Trail Making Test	Estimation of physical activity levels with questionnaire: Lüdenscheid Activity Questionnaire	= speed of processing, attention, and memory in physically active group. ↑ Stroop task performance in physically active group = CNV amplitude in physically active group ↑ EEG-ERP: N2 and N450 amplitude in physically active group
Schättin, 2016 (RCS) [38]	Healthy men and women mean age 79.2 ± 7.3 years. Exergame: n = 13 Balance: n = 14	EEG spectral power	Executive Functions: Test for Attentional Performance	Exergame or balance Training: 24 sessions for 8 to 10 weeks	↑ Executive Functions in exergame group ↓ Theta relative power exergame group
Schättin, 2018 (OBS) [39]	Healthy men and women mean age 73.3 ± 5.9 years. Single group: n = 36	EEG spectral power	Attention: Test of Attentional Performance	Estimation of physical activity levels with questionnaire: Physical Activity Questionnaire 50+	= attention in physically active group ↑ EEG power in the alpha frequency in physically active group
Gajewski, 2018 (RCS) [40]	Healthy men and women aged 65–88 years. Physical training: n = 37 Control: n = 40	EEG-ERP: P3a, P3b	Memory: Verbal Learning and Memory Test, Word Fluency Test, Rey-Osterrieth Complex Figure Working memory: Digit-Span Test	Physical training: cardiovascular, aerobic, and strength exercises. For 4 months, two times per week and 90 min per session	= memory in physically active group = EEG-ERP: P3a, P3b amplitude in physically active group
Schättin, 2019 (RCS) [41]	Healthy men and women mean age 69.4 ± 4.6 years. Single group: n = 17	EEG: response-locked potentials	Attention: Test of Attentional Performance	Exergame Training: 30 sessions for 10 weeks	= Attention in physically active group = EEG: response-locked potentials in physically active group
Adcock, 2020 (INT) [42]	Healthy female 71.4 ± 5.8 years. Single group: n = 19	EEG: power spectral density	Attention: Test of Attentional Performance	Exergame Training: 21 sessions for 7 weeks	↑Attention in physically active group = EEG: alpha power spectral density in physically active group

OBS: Observational study; INT: intervention study; RCS: Randomized Controlled Study; RCT: Randomized Control Trial; EEG-ERP: Event Related Potentials. ↑ indicates increase, ↓ indicates decrease, = indicates no change.

## 4. Discussion

The present systematic review aimed to identify, evaluate, and summarize findings from human studies that provide compelling evidence of changes in EEG activity and cognition associated with PA in older adults. In this regard, the PICO research question considered the following elements: Population—older adults; Intervention—physical activity; Comparison—control or sedentary group, or pre-post measure; and Outcome measure—effect on EEG and cognition. Evidence from the included studies suggests that moderate PA interventions, such as exergames and brisk walking, improve cognition and EEG parameters [36,38,42], whereas light PA, such as yoga or aerobic interventions, revealed no changes in cognition or EEG measures [35,40]. Furthermore, in studies using self-reported PA, one reported improvements in both cognitive measures and EEG parameters [23] while two others reported no changes in cognitive measures but improvements in EEG measures [37,39]. These inconsistencies may be attributed to the self-reporting measure of PA. In this regard, PA interventions in older adults with type 2 diabetes and cognitive impairment have shown that both high-intensity/low-volume and low-intensity/high-volume PA improved cognitive performance compared with a control group, and that visuospatial memory was better in the high-intensity training group compared with the low-intensity and control groups [46]. However, Sanders et al. [47] reported no significant differences in cognitive function between low- and high-intensity aerobic or resistance exercise, nor when compared to a control group, in older adults with dementia. This lack of significant differences between high- and low-intensity exercise with regard to cognitive outcomes may be explained by the individual conditions of the participants, as well as the type and intensity of the PA. Evidence from a systematic review [48] evaluating the moderating effects of exercise intensity in healthy subjects aged 18 years or older indicated that moderate acute resistance exercise consistently shows beneficial effects on inhibitory control, while light or vigorous intensity resistance exercise yielded benefits in about 50% of cases, aligning with the results found in the present systematic review.

PA in older adults should be considered as one of the main strategies to promote health and prevent diseases, especially cognitive impairment [5,49,50]. In this regard, evidence suggests that older adults with higher levels of PA tend to have higher cognitive reserve scores and experience less cognitive impairment [16,18]. Iso-Markku et al. [51] further demonstrated the protective effects of PA, highlighting the buffering role in the presence of mild cognitive impairment. Recently, Makino et al. [52] assessed PA habits at different stages (early, middle, and late adulthood) and their correlation with cognitive function in old age, indicating that lifelong PA is associated with a lower likelihood of mild cognitive impairment. In addition, cognitive function in older adults has been shown to improve with aerobic exercise over a 5-year period [53]. One of the crucial elements found in the present systematic review that affects the results is the typology of the subjects, i.e., whether they are physically active or not, not only throughout their lives but also at the time of the evaluation. In this sense, Domingos et al. [54] compared a group of athletes with non-physically active subjects and concluded that neurofeedback training increased the relative amplitude of the bands in the group of non-athletes; however, only the group of athletes showed improvements in the performance tests performed after 12 sessions of neurofeedback training. These findings, along with those discussed in this review, suggest that PA may be a potential strategic intervention to prevent cognitive decline with aging. In this regard, an interesting line of future exploration may be the outcome of an active lifestyle from an early age as a preservation factor for cognition with advanced age.

Studies have suggested that EEG shows positive activity changes in prefrontal areas during the performance of a cognitive task in older adults [26]. In this context, PA interventions that combined cardiovascular and muscle-strengthening exercises over a longer time showed greater benefits in terms of both cognitive function and the preservation of age-related neurological effects, according to EEG evaluations, as interventions sustained for more than 6 weeks resulted in patient improvements in functions such as short- and long-term memory, alertness, and processing speed. In the same vein, the analysis showed that the continued practice of PA can lead to improvements at the cognitive level, with a direct positive impact on the health of older adults, suggesting that this may be a potential therapeutic target to prevent cognitive decline and the neurophysiological changes associated with aging [36,37,38,39]. Additionally, evidence indicates that combined aerobic and resistance training and exercise interventions in healthy, older adults improve neuroplasticity, while low-intensity exercise does not have this effect. Furthermore, high-intensity exercise was shown to improve neuroplasticity markers more than low-intensity exercise [55]. In this regard, it is important to note that brain plasticity due to PA can be detected at the cognitive level in humans [56], and that it can be measured directly with EEG, which provides direct measurements of the functional neuroelectric activity that underlies neuroplasticity [57].

In terms of the EEG modifications associated with PA interventions, the results indicated that PA is associated with changes such as higher multiscale entropy EEG, lower theta power, higher alpha power, and higher beta power [23,38,39], findings that indicate that PA helps reduce some of the EEG changes reported as normal signs of aging [29,30]. Additionally, the results indicated that long term PA is associated with a shorter latency for N2 and P2 and P3 ERPs, greater amplitude in N2, and more negative amplitude in N450 ERPs in frontocentral regions (e.g., Cz, Pz electrodes) [36,37,40]. Furthermore, evidence shows that a physically active lifestyle is related to higher alpha frequency power activity in prefrontal-located EEG electrodes [39]. These findings are consistent with changes in brain activity in the frontal and posterior areas of the PASA healthy aging model [26,27].

In this regard, it has been reported that in older adults with mild cognitive impairment, aerobic and resistance training interventions for 24 weeks significantly reduced theta/beta ratio, which reflects improvements in attention-related functions, in the temporal and parietal lobes, and in the delta/alpha ratio, which is associated with cognitive impairment, in the frontal, temporal, and parietal areas [58]. Furthermore, Dimitrova et al. [59] emphasized that just 20 min of moderate exercise increases the EEG amplitude from 320 to 700 ms at the Cz site and enhances executive task performance in older adults. Additionally, in healthy, older adults, an acute 25-min exercise intervention resulted in greater task-related power decreases in the beta band, an indicator of active motor processing, in contralateral frontal brain areas compared to a control condition, indicating enhanced cortical activation and motor memory consolidation [60]. There is also evidence that a single session of resistance training significantly reduces the conflict-related neural activity N2b ERP local peak amplitude in congruent and incongruent conditions and improves information processing [61]. Moreover, evidence indicates that in older adults, acute light and moderate-intensity exercise has an effect on ERP amplitude and latency, i.e., reducing P3 latency and increasing P3 amplitude, whereas chronic moderate-intensity exercise was reported to reduce N2 and P2 latency [62]. These findings indicate that, regardless of the duration of the intervention, PA has beneficial effects on the brain activity of older adults.

In summary, research suggests a consistent pattern of positive cognitive and EEG changes associated with PA interventions. The results indicate that both aerobic and resistance training interventions show promising effects on attention-related functions, cognitive impairment, and executive task performance. Furthermore, understanding the moderating effects of PA intensity is critical, as moderate-intensity PA has consistently demonstrated benefits in terms of cognitive function, while the effects of light- or vigorous-intensity PA varied across studies. Overall, the findings demonstrate the multifaceted effects of PA on cognitive and neural processes and highlight the potential short- and long-term benefits of PA on brain function, providing valuable insights into its role in promoting cognitive health and brain resilience in older adults.

## 5. Conclusions

In this review, we systematically analyzed the benefits of PA in healthy, older adults in terms of its effects on EEG and cognition. The results of the studies in the review were that the effects of PA are different in low and high intensity exercise for both cognitive function and EEG. This suggests that PA may be a potential therapeutic measure to prevent cognitive decline and the neurophysiological changes associated with aging. Moreover, the exercise-induced neuroplasticity that underlies improvements in cognitive function in healthy older adults can be identified based on EEG activity. The results of this systematic review highlight the importance of taking into account the specific type and intensity of PA when designing interventions to improve cognitive function and brain health in older adults.
